# SPIE Computer-Aided Diagnosis conference anniversary review

**DOI:** 10.1117/1.JMI.9.S1.012208

**Published:** 2022-05-19

**Authors:** Ronald M. Summers, Maryellen L. Giger

**Affiliations:** a National Institutes of Health, Radiology and Imaging Sciences, Clinical Center, Bethesda, Maryland, United States; b University of Chicago, Department of Radiology and Committee on Medical Physics, Chicago, Illinois, United States

**Keywords:** lung, breast, colon, heart, COVID-19, deep learning

## Abstract

The SPIE Computer-Aided Diagnosis conference has been held for 16 consecutive years at the annual SPIE Medical Imaging symposium. The conference remains vibrant, with a core group of submitters as well as new submitters and attendees each year. Recent developments include a marked shift in submissions relating to the artificial intelligence revolution in medical image analysis. This review describes the topics and trends observed in research presented at the Computer-Aided Diagnosis conference as part of the 50th-anniversary celebration of SPIE Medical Imaging.

## 1 Introduction

The Computer-Aided Diagnosis (CAD) conference at the annual SPIE Medical Imaging symposium reaches its 16th anniversary in 2022. An outgrowth of the tremendous interest in computer-aided diagnosis in biomedical imaging in the 1990s and early 2000s led to the creation of this separate conference. Prior to that time, computer-aided diagnosis papers were included in the Image Processing, Biomedical Applications, Picture Archiving and Communication Systems, and Perception conferences, all held within the annual SPIE Medical Imaging symposium. There are many commonalities between the Image Processing and CAD conferences at the annual Medical Imaging meeting. However, the evolution of the CAD conference from the Imaging Processing conference was the recognition that additional aspects of the overall research task included a greater need for both clinical input (on both the clinical question and the clinical outcomes) and a systems approach to the detection (localization) and diagnosis (classification) problems. Interestingly, many of the very early “firsts” in CAD were presented in the Imaging Processing conference prior to the launch of the CAD conference.

**Fig. 1:**
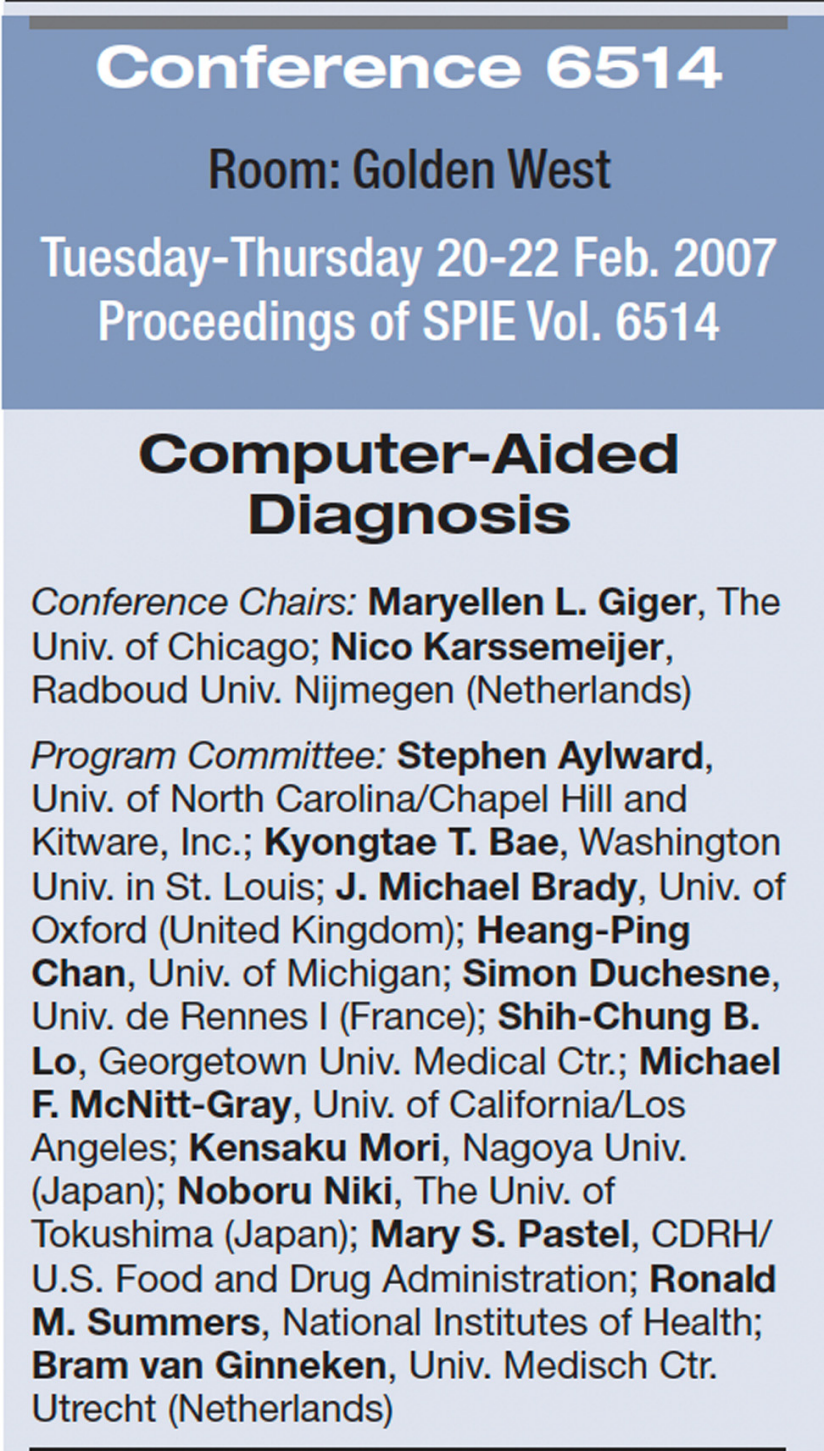
Extract from the 2007 SPIE Medical Imaging program showing the inaugural CAD conference program committee.

The inaugural CAD conference was held in San Diego, California, in 2007 and spanned 3 days (Fig. 1). The conference was chaired by Maryellen Giger and Nico Karssemeijer. There were 12 program committee members with international representation including the United States, United Kingdom, France, Japan, and the Netherlands, and hailing from academia, government agencies (such as NIH and FDA), industry, and clinical practice. Over the years, new program committee members have been added. By 2022, the committee had grown to 48 members, including the two conference chairs, with international representation including the United States, Brazil, China, France, Germany, Israel, Japan, Korea, the Netherlands, and the United Kingdom. The chairs and cochairs for each year's CAD conference are listed in Table 1.

**Fig. 2:**
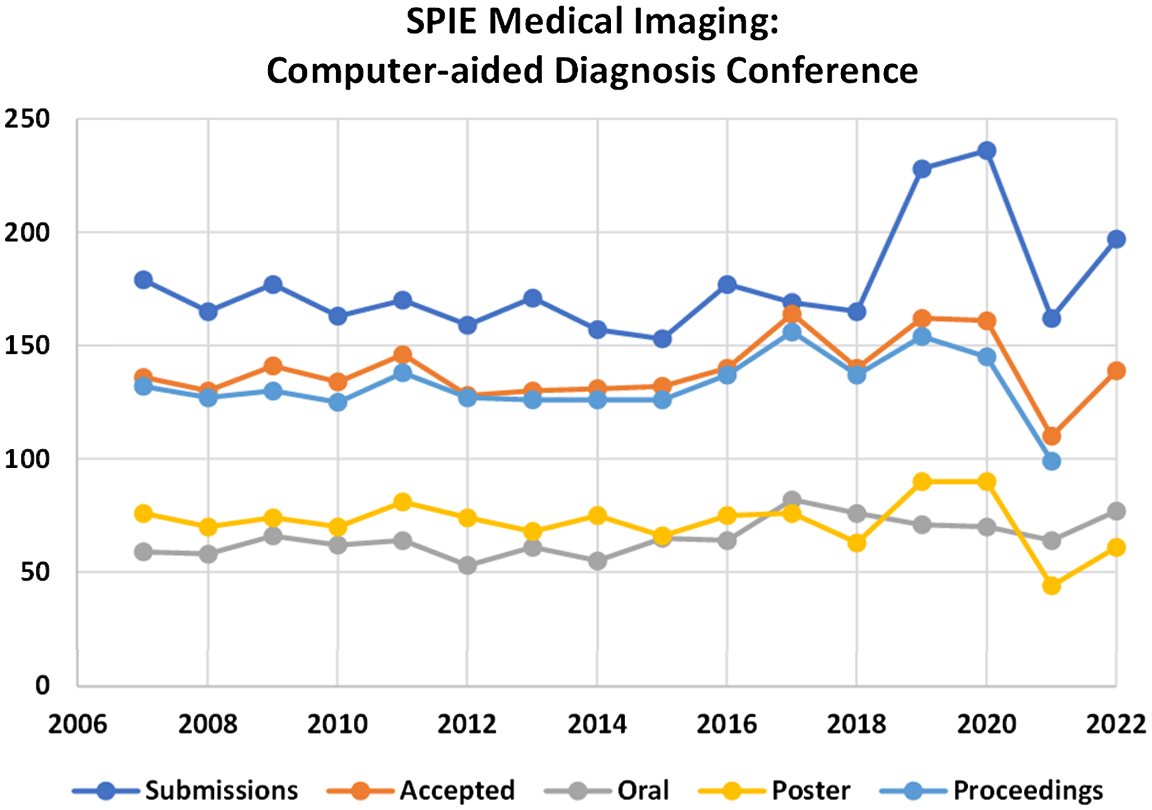
Statistics for the SPIE Medical Imaging Computer-Aided Diagnosis conference. Numbers of submissions, accepted, oral, and poster presentations and published proceedings articles are shown. Data courtesy of SPIE.

At the inaugural conference in 2007, the oral sessions were divided into 12 separate sessions. The section topics were mammogram analysis, CT colon, a keynote session, pathology imaging, thoracic CT, MRI applications, CT lung nodules, breast tomosynthesis, cardiac/new applications, breast imaging, and thoracic/skeletal imaging. The conference had 179 submissions and 136 accepted papers. These were divided into 1 keynote, 59 oral, and 77 poster exhibits. The conference proceedings included 132 published full papers. In 2021, the oral sessions were divided into 13 separate sessions. The topics included a keynote session, lung (three separate sessions), breast (two sessions), abdomen (two sessions), cardiovascular and ophthalmology, musculoskeletal, pediatric/fetal applications, methodology, and neuroradiology including head and neck. The conference had 162 submissions and 110 accepted papers. These were divided into 64 oral and 44 poster exhibits. The conference proceedings included 99 published full papers and 102 presentations. Figure 2 shows statistics of submissions, acceptances, oral and poster presentations, and publications. The acceptance rate averaged 79% (range 68% to 97%).

The CAD conference included a number of special sessions, frequently co-organized with one of the other SPIE Medical Imaging conferences. Many of the special sessions included panel discussions. Special sessions included Critical Issues in Adapting CAD into Clinical Practice (2008), Digital Pathology (2012), Challenges in CAD Development and Commercialization (2013), CAD Successes and Failures (2014), CAD Grand Challenge—Present and Future (2015), SPIE/IFCARS Joint Workshop on Information Management, Systems Integration, Standards, and Approval Issues for the Digital Operating Room (2016 and 2017), and Simulated Tumor Board: Brain and Breast (2020). These panel discussions, such as the 2020 Simulated Tumor Board, often included clinicians, beyond the regular scientific and technical attendees of SPIE MI, to expand the clinical knowledge base of the CAD researchers, many of whom might not have access to clinicians.

Many of the other CAD conference special sessions included Grand Challenges with their discussions and outcomes including the SPIE-AAPM-NCI Lung Nodule Classification Challenge (LUNGx) (2015), SPIE-AAPM-NCI CAD Grand Challenges: Paving the Way for Imaging in the Era of Precision Medicine (2016), PROSTATEx Challenge and Digital Mammography DREAM Challenge (2017), PROSTATEx Lessons Learned and 2019 Challenge (2018), and BreastPathQ: Cancer Cellularity Challenge (2019).

Keynote speakers are highlights of the annual conference. The conference’s inaugural keynote speaker in 2007 was Robert F. Wagner from the FDA. His keynote topic was “Computer-aided diagnosis and the general bioinformatics problem.” The keynote speakers and topics presented are shown in Table 2.

**Table 1:** Conference cochairs.

Year	Chair	Cochair
2007	Maryellen L. Giger, The Univ. of Chicago (United States)	Nico Karssemeijer, Radboud Univ. Nijmegen Medical Ctr. (The Netherlands)
2008	Maryellen L. Giger, The Univ. of Chicago (United States)	Nico Karssemeijer, Radboud Univ. Nijmegen Medical Ctr. (The Netherlands)
2009	Nico Karssemeijer, Radboud Univ. Nijmegen Medical Ctr. (The Netherlands)	Maryellen L. Giger, The Univ. of Chicago (United States)
2010	Nico Karssemeijer, Radboud Univ. Nijmegen Medical Ctr. (The Netherlands)	Ronald M. Summers, National Institutes of Health (United States)
2011	Ronald M. Summers, National Institutes of Health (United States)	Bram van Ginneken, Univ. Medical Ctr. Utrecht (The Netherlands)
2012	Bram van Ginneken, Radboud Univ. Nijmegen (The Netherlands)	Carol L. Novak, Siemens Corporate Research (United States)
2013	Carol L. Novak, Siemens Corporate Research & Technology (United States)	Stephen Aylward, Kitware, Inc. (United States)
2014	Stephen Aylward, Kitware, Inc. (United States)	Lubomir M. Hadjiiski, Univ. of Michigan Health System (United States)
2015	Lubomir M. Hadjiiski, Univ. of Michigan Health System (United States)	Georgia D. Tourassi, Oak Ridge National Lab. (United States)
2016	Georgia D. Tourassi, Oak Ridge National Lab. (United States)	Samuel G. Armato III, The Univ. of Chicago (United States)
2017	Samuel G. Armato III, The Univ. of Chicago (United States)	Nicholas A. Petrick, U.S. Food and Drug Administration (United States)
2018	Nicholas A. Petrick, U.S. Food and Drug Administration (United States)	Kensaku Mori, Nagoya Univ. (Japan)
2019	Kensaku Mori, Nagoya Univ. (Japan)	Horst K. Hahn, Fraunhofer MEVIS (Germany)
2020	Horst K. Hahn, Fraunhofer MEVIS (Germany), Jacobs Univ. Bremen (Germany)	Maciej A. Mazurowski, Duke Univ. (United States)
2021	Maciej A. Mazurowski, Duke Univ. (United States)	Karen Drukker, The Univ. of Chicago (United States)
2022	Karen Drukker, The Univ. of Chicago (United States)	Khan M. Iftekharuddin, Old Dominion Univ. (United States)

**Table 2:** Keynote speakers and topics.

Year	Speaker	Topic
2007	Robert F. Wagner, U.S. Food and Drug Administration (United States)	Computer-aided diagnosis and the general bioinformatics problem
2008	Heinz-Otto Peitgen, MeVis Research GmbH (Germany) and Florida Atlantic Univ. (United States)	Clinical relevance of computer-aided diagnosis and visualization
2009	Kyle J. Myers, U.S. Food and Drug Administration. (United States)	(Joint Keynote Session) Medical Imaging and Radiological Health: Contributions of Dr. Robert F. Wagner
2010	Kunio Doi, The Univ. of Chicago (United States)	Computer-aided diagnosis in medical imaging: achievements and challenges
2011	Heang-Ping Chan, Univ. of Michigan Health System (United States)	CAD: past, present, and future
2012	Michael D. Abramoff, The Univ. of Iowa Hospitals and Clinics and Univ. of Iowa (United States)	Automated detection of retinal disease: when Moore’s law meets Baumol’s cost disease
2013	Panel discussion	Challenges in CAD development and commercialization
2014	Nico Karssemeijer, Radboud Univ. Nijmegen Medical Ctr. (Netherlands); Eliot L. Siegel, Univ. of Maryland Medical Ctr. (United States)	(Joint Keynote Session) Opportunities and challenges for diagnostic decision support systems, and rethinking CAD for the future: a clinical perspective
2015	Tanveer F. Syeda-Mahmood, IBM Research—Almaden (United States)	Role of machine learning in clinical decision support
2016	Hugo Aerts, Dana-Farber Cancer Institute (United States) and Brigham and Women’s Hospital (United States) and Harvard Medical School (United States)	Radiomics: there is more than meets the eye in medical imaging
2017	Kyle J. Myers, U.S. Food and Drug Administration (United States)	FDA’s role in the innovation and evaluation of evolving CAD solutions
2018	Gustavo A. Stolovitzky, IBM Thomas J. Watson Research Ctr. (United States) and Icahn School of Medicine at Mount Sinai (United States)	Crowdsourcing Biomedical Research: Leveraging Communities as Innovation Engines
2019	Bernardino Romera-Paredes, Google DeepMind (United Kingdom)	The U-net and its impact on medical imaging
2020	Jonathan I. Wiener, Boca Radiology Group and FAU Medical School (United States)	Will AI make me a better doctor?
2021	Saurabh Jha, Univ. of Pennsylvania (United States)	Decoding radiology: a brief history
2022	Jayashree Kalpathy-Cramer, MGH/Harvard Medical School (United States)	Deep learning in medical imaging: a practical guide to opportunities and challenges

Live demonstrations, initiated by the CAD conference at the SPIE Medical Imaging meeting, are a popular session at the CAD conference. Begun at the inaugural CAD conference in 2007 and led by Maryellen L. Giger, The Univ. of Chicago (United States); Nico Karssemeijer, Radboud, Univ. Nijmegen (The Netherlands); and Michael F. McNitt-Gray, Univ. of California/Los Angeles (United States), live hands-on demonstrations continued annually thereafter. Organizers of the live demonstrations in later years included Bram van Ginneken, Univ. Medisch Ctr. Utrecht (The Netherlands); Stephen R. Aylward, Kitware, Inc. (United States); Heang-Ping Chan, Univ. of Michigan (United States); Horst Hahn, Fraunhofer MEVIS, (Germany); Lubomir Hadjiiski, Univ. of Michigan Health System (United States); and Karen Drukker, Univ. of Chicago (United States). Attendees vote for their favorite demonstration each year and awards are given for the highest vote-getter.

The top contributors to the CAD conference are shown in Tables 3 and 4. Over the years, the most prolific contributor to the CAD conference has been Heang-Ping Chan, PhD, from the University of Michigan. The top contributing institution has been the University of Chicago.

**Table 3:** Top contributors to proceeding papers from the SPIE Medical Imaging CAD conferences.

Author	Number of published proceeding papers from the SPIE Medical Imaging CAD conference
Heang-Ping Chan	97
Lubomir M. Hadjiiski	89
Chuan Zhou	59
Hiroshi Fujita	57
Jun Wei	54
Maryellen L. Giger	49
Bin Zheng	42
Ronald M. Summers	42
Kensaku Mori	37
Berkman Sahiner	35

Note: Numbers of articles published in the conference proceedings and co-authored by the given author. Search terms (Date of search October 29, 2021; only includes published proceedings articles, not abstracts that did not lead to a published proceedings article): scholarly works (1974) = [SPIE AND (Medical AND Imaging)] AND Source Title: (computer-aided AND diagnosis).^1^

**Table 4:** Top contributing institutions to proceeding papers from the SPIE Medical Imaging CAD conferences.

Authors’ institution	Number of published proceeding papers from the SPIE Medical Imaging CAD conference
University of Chicago	107
University of Michigan	102
National Institutes of Health	77
Gifu University	57
Rabdoud University	55
Duke University	52
University of Pennsylvania	50
Siemens	48
Harvard University	45
Nagoya University	42

Note: Search terms (Date of search October 29, 2021; only includes published proceedings articles, not abstracts that did not lead to a published proceedings article): Scholarly Works (1974) = [SPIE AND (Medical AND Imaging)] AND source title: (Computer-aided AND Diagnosis).^1^

The most downloaded papers of all time and from 2021 are shown in Tables 5 and 6, respectively. The all-time most downloaded papers cover a variety of topics including breast, brain, cardiac, and prostate imaging. The most downloaded papers from 2021 emphasized deep learning and COVID-19.

**Table 5:** Top 10 CAD proceedings paper downloads, 2007 to 2021.

Paper	Downloads
Wu S. D. et al. (2012), Fully automated chest wall line segmentation in breast MRI by using context information^2^	4030
Koenrades M. A. et al. (2017), Validation of an image registration and segmentation method to measure stent graft motion on ECG-gated CT using a physical dynamic stent graft model^3^	2860
Wegmayr V. et al. (2018), Classification of brain MRI with big data and deep 3D convolutional neural networks^4^	1913
Bar Y. et al. (2015), Deep learning with non-medical training used for chest pathology identification^5^	1482
Sun W. Q. et al. (2016), Computer aided lung cancer diagnosis with deep learning algorithms^6^	1454
Ramachandran S. S. et al. (2018), Using YOLO based deep learning network for real time detection and localization of lung nodules from low dose CT scans^7^	1383
Jnawali K. et al. (2018), Deep 3D convolution neural network for CT brain hemorrhage classification^8^	1238
Wei Q. et al. (2018), Anomaly detection for medical images based on a oneclass classification^9^	1161
Liu S. F. et al. (2017), Prostate cancer diagnosis using deep learning with 3D multiparametric MRI^10^	817
Tsehay Y. K. et al. (2017), Convolutional neural network based deep-learning architecture for prostate cancer detection on multiparametric magnetic resonance images^11^	723

Note: Data as of January 10, 2022, courtesy of SPIE.

**Table 6:** Top 10 CAD proceedings paper downloads from 2021 (Vol. 11597).

Paper	Downloads
Heidari M. et al., Detecting COVID-19 infected pneumonia from x-ray images using a deep learning model with image preprocessing algorithm^12^	340
Paul R. et al., Deep radiomics: deep learning on radiomics texture images^13^	255
Sriker D. et al., Improved segmentation by adversarial U-Net^14^	198
Hu Q. Y. et al., Role of standard and soft tissue chest radiography images in COVID-19 diagnosis using deep learning^15^	195
Pan M. Q. et al., Deep learning-based aggressive progression prediction from CT images of hepatocellular carcinoma^16^	182
Prasad P. J. R. et al., Modifying U-Net for small dataset: a simplified U-Net version for liver parenchyma segmentation^17^	175
Moreau N. et al., Comparison between threshold-based and deep learning-based bone segmentation on whole-body CT images^18^	159
Luna J. M. et al., Radiomic features predict local failure-free survival in stage III NSCLC adenocarcinoma treated with chemoradiation^19^	159
Vu Y. N. T. et al., An improved mammography malignancy model with selfsupervised learning^20^	159
Agarwal C. et al., CoroNet: a deep network architecture for enhanced identification of COVID-19 from chest x-ray images^21^	157

Note: Data as of January 10, 2022, courtesy of SPIE.

The sessions at the CAD conference are typically organized by body organ rather than by methodology. Lung and breast have been two consistently presented areas throughout the life of the CAD conference. Other frequent topics include the abdomen, colon, cardiac and vascular, musculoskeletal, radiomics, deep learning, brain, head and neck, eye, and pathology imaging (which later became the separate Digital Pathology conference). As COVID-19 arose, it also became a topic within the CAD conference.

While artificial neural networks, including deep learning with early versions of convolutional neural networks, had been included in SPIE CAD presentations since the mid-1990s, deep learning became a major focus in about 2016 and became the preeminent method of machine learning in subsequent years.

In the next section, we review some of the topics covered during the life of the CAD conference. Because of the large number of oral and poster presentations over the years, only a small number of representative examples can be listed.

Lung nodule analysis has been a consistent theme throughout the history of the SPIE Medical Imaging symposium and was a major theme that transferred from the Image Processing conference to the CAD conference.^22^^–^^24^ The Lung Image Database Consortium had several early papers.^25^ Lung nodule phantoms were a popular theme.^26^ Temporal analysis of lung disease also attracted attention.^27^ Other thoracic disease topics of recurrent interest included chronic obstructive pulmonary disease (COPD) and emphysema, diffuse lung parenchymal disease, lung cancer, pneumothorax detection, pneumoconiosis, tuberculosis, pleural effusions, and pulmonary embolism detection.^28^^–^^35^ Pulmonary patterns including texture analysis were a popular topic in 2010.^36^ In 2016, texture analysis was combined with deep learning.^37^ Chest radiograph diagnosis was notably enhanced with deep learning thereafter.^38^^–^^41^ Other notable topics included H1N1 pneumonia and population screening using chest radiography.^42^^,^^43^ Anatomic topics included interlobar fissure detection, mediastinal lymph node station mapping, airway analysis, and guidance for interventions.^44^^–^^48^ Introduction of thoracic low-dose CT (LDCT) led to the development of AI for emphysema, coronary artery calcifications, and osteoporosis.^49^^,^^50^ As COVID-19 arose with its presentation on chest radiographs and thoracic CTs, AI methods for COVID became a part of the CAD conference presentations.^15^^,^^51^

With the continuing rise in mammographic screening and multimodality breast diagnosis computer vision and machine learning systems, it is not surprising that breast has been a mainstay in the CAD conference. Many of the presenters on breast CAD had previously submitted to the image processing conference. Beyond full-field digital mammograms and breast ultrasound, CAD on breast tomosynthesis was an early topic for emerging technology.^52^^–^^54^ Other breast imaging technologies and topics with CAD applications included dynamic breast MRI, utilization of multiple views, lesion segmentation and classification, breast segmentation and density assessment, predictive models for cancer risk assessment, dedicated breast CT, 3D ultrasound, and breast cancer diagnosis with deep learning.^55^^–^^62^ In addition, AI methods for assessing prognosis and response to therapy have been presented.^63^

Abdominal imaging with a focus on bowel and liver was a frequent topic. Automated colonic polyp detection, classification, and measurement of CTC with or without traditional cathartic colon cleansing were popular topics in the early years of the conference before CT colonography became a mainstream clinical technique.^64^^–^^69^ Colon and colonic polyp analysis further included dual-energy CT colonography, taeniae coli detection, supine-prone colonic polyp registration, colitis detection, and colonoscopy video analysis.^70^^–^^74^ Other abdominal topics have included bladder segmentation, small bowel analysis including segmentation and Crohn disease detection, endoscopic image analysis for polyps and cancers, liver organ and lesion segmentation, liver elastography, kidney segmentation, renal calculi detection, pancreas segmentation, pancreatic cyst classification, and uterine and placental segmentation.^75^^–^^85^

Prostate MRI analysis, including whole gland segmentation, cancerous and noncancerous lesion detection and classification, and multiparametric and dynamic contrast-enhanced prostate MRI analysis, was also presented as part of various topics.^11^^,^^86^^–^^91^ Occasional presentations have focused on CAD in other oncologic diseases including assessment of lymphadenopathy, cervical cancer, esophageal cancer, pancreatic tumors, and multiple myeloma.^92^^–^^98^

CAD of cardiac and vascular imaging included coronary artery calcium scoring with deep learning, coronary artery detection, and stenosis analysis on angiography and CT, intravascular OCT, cardiomegaly assessment, and cardiac wall and chamber assessment.^99^^–^^104^ Atherosclerotic disease outside the heart was also assessed.^105^^,^^106^

CAD of brain imaging included detection, segmentation, and classification of brain tumors, Alzheimer’s dementia, neonatal brain analysis, stroke outcome prediction, radiogenomics of glioblastoma, intracranial hemorrhage and aneurysms, hydrocephalus diagnosis, glioma mutation assessment, and traumatic brain injury.^8^^,^^107^^–^^116^ A notable topic was the detection of head malformations in craniosynostosis from 3D photographs.^117^

CAD approaches in musculoskeletal imaging have focused on the spine and appendicular skeleton and the muscles and joints. Topics included fracture and metastases detection, bone quality, vertebral segmentation, spinal and neural foraminal stenosis detection, scoliosis and intervertebral disk degeneration assessment, localization of the epiphyses, automated bone mineral densitometry, osteoporosis, osteolysis, and muscle segmentation including analysis of the psoas muscles in amyotrophic lateral sclerosis.^118^^–^^127^

Analysis of pathology images was initially in the CAD conference including cytologic and histologic automated diagnosis, and multispectral fluorescence microscopy.^128^^,^^129^ However, now with the digital pathology conference at the SPIE Medical Imaging meeting, most papers have moved there.

CAD of ophthalmological imaging has included analysis of images for diabetic retinopathy, retinal vascular analysis including microaneurysm detection, macular degeneration, malaria retinopathy, retinal cone photoreceptor detection, and retinopathy of prematurity.^130^^–^^136^

Radiomics, a more recent term for the human-engineered features extracted in many CAD algorithms, was first included as a session topic in 2016. Radiomics topics have included associations between breast MRI features and gene expression, associations of radiomics features with acquisition-related parameters such as interscanner variations and MR magnet strengths, harmonization methods, and prediction of molecular subtypes of pediatric medulloblastoma, as well as assessment of the effect of variations in texture software packages on algorithm performance and robustness.^137^^–^^141^

Other topics have included multiorgan segmentation, CAD methodology, CAD software, dental applications including arthritis of the temporomandibular joint (TMJ), and analysis of chronic wound, skin lesion, and eardrum images.^142^^–^^150^ Endocrine analysis included thyroid and parotid gland segmentation.^151^^,^^152^ Surgical applications included detection of retained foreign bodies.^153^

With 2085 accepted papers and 1985 published proceedings articles through 2021, the SPIE Medical Imaging CAD conference continues to thrive. The deep learning revolution in medical image processing has greatly contributed to this growth. It is expected that deep learning will continue to be one of the main drivers of scientific advances in computer-aided diagnosis over the next 5 to 10 years.

The authors thank the many program committee members, conference chairs, session chairs, and authors whose ongoing participation contributed to the success of the CAD conference.
